# *In Vitro* and *In Vivo* Studies of Titanium Dioxide Nanoparticles with Galactose Coating as a Prospective Drug Carrier

**DOI:** 10.1021/acsomega.4c02232

**Published:** 2024-08-15

**Authors:** Jolanta Pulit-Prociak, Olga Długosz, Anita Staroń, Dominik Domagała, Krzysztof Pociecha, Mikołaj Grabowski, Michał Zielina, Marcin Banach

**Affiliations:** † Faculty of Chemical Engineering and Technology, Cracow University of Technology, Warszawska 24, Cracow 31-155, Poland; ‡ Faculty of Food Technology, University of Agriculture in Krakow, Balicka 122, Cracow 30-149, Poland; § Faculty of Pharmacy, Jagiellonian University, Medyczna 9, Cracow 30-688, Poland; ∥ Faculty of Environmental Engineering and Energy, Cracow University of Technology, Warszawska 24, Cracow 31-155, Poland

## Abstract

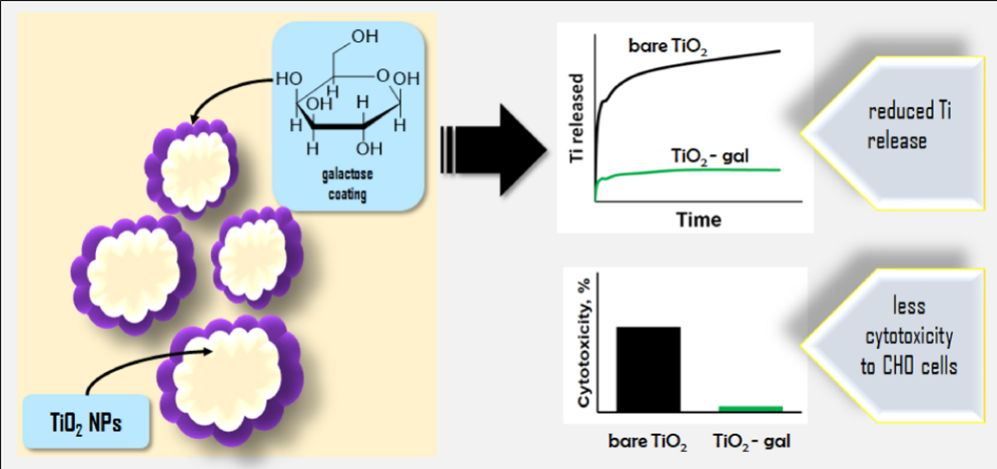

In today’s medicine, progress often depends on new products with special qualities. Nanotechnology focuses on the creation of materials tailored to fulfill specific therapeutic requirements. This study aims to elucidate the potential of nanoparticles, particularly titanium dioxide nanoparticles, as carriers for pharmaceutical agents. To mitigate the release of potentially harmful titanium ions from the carrier’s surface, modifications were implemented. In the initial phase, titanium dioxide, nanoparticles were obtained based on the sol–gel method, and their surfaces were coated with galactose. Characterization of these materials encompassed analysis of the particle size, specific surface area, microscopic morphology, and titanium ion release. Additionally, drug release profiles, particularly those of tadalafil, were investigated. *In vitro* assessments were conducted to evaluate the cytotoxic and mutagenic effects of the developed materials on CHO cells. The findings revealed a reduction in titanium ion release from the modified carrier compared to its unmodified counterpart. Pharmacokinetic studies in rats demonstrated enhanced absorption of the drug when the drug was delivered using the modified carrier. The synthesized materials exhibited high purity and favorable surface properties conducive to effective drug–carrier interactions. The results suggest that the modified titanium dioxide nanoparticles hold promise as efficient drug delivery vehicles in biomedical applications.

## 1 Introduction

The most frequently discussed issue in the literature regarding the use of nanomaterials and nanoparticles in pharmacy and medicine is their use in targeted therapy. Promising results are being achieved in cases of patients with cancerous diseases, where nanoparticles play a crucial role in delivering the active substance to affected cells and tissues. Due to their cytotoxicity toward cancer cells, scientists are not confining their use to mere drug carriers (monofunctional nanoparticles).^1^ Research is underway on multifunctional nanoparticles capable of integrating various functions within their core or surface, aiming to synergistically maximize anticancer activity. It is increasingly evident that effective therapeutic approaches require multiple drugs and targets. Multifunctional nanoparticles can be created using two main categories of nanomaterials: organic (such as micelles, liposomes, nanogels, and dendrimers)^2^ and inorganic materials (like superparamagnetic iron oxide (SPIO), gold, quantum dots (QD), and lanthanide ions).^3^ Titanium dioxide nanoparticles have emerged as a new generation of advanced materials due to their optical, dielectric, and photocatalytic properties stemming from their small size.^4^ They find wide application in industrial and consumer goods owing to their robust catalytic activity, with known bactericidal properties.^5,6^ In recent years, titanium dioxide nanoparticles have accounted for nearly 5% of the total production of titanium dioxide. Combining titanium dioxide nanoparticles with various molecules, antibodies or polymers has shown promising photocytotoxic effects against cancer cells and microbes, revealing potential application in photodynamic therapy.^7^

The additional silica dioxide coating influences their functionality with a suitably thick silica dioxide layer ensuring optimal preservation of the photodynamic properties and enhancing biocompatibility. Forming a composite with chitosan may expand its current use toward wound treatment.^8^ Ongoing research focuses on the modification of titanium dioxide nanoparticles to enhance their sensitivity in photodynamic therapy, particularly toward visible light, therby bolstering their utility in medical applications.^9^

The uncontrolled distribution and unregulated release of active substances in traditional drug delivery systems have driven the development of intelligent drug delivery systems based on nanocarriers. These systems exhibit the capacity to transport drugs to targeted sites with reduced dosing frequency and precise spatial control.^10^ The utilization of nanoparticles for drug delivery can augment the amount of drug reaching the intended site, subsequently diminishing the necessary drug doses and thereby mitigating toxic side effects.^11^ To realize the clinical potential of newly designed nanoparticle-based carriers, several critical elements must be taken into account. First, the design of these nanomaterials should focus on essential factors such as sufficient biocompatibility, biodegradability, and stability in physiological conditions, high drug-loading capacity, and low toxicity. Furthermore, aside from the fundamental requirements of safety and therapeutic performance, the feasibility of big-scale production is also a requisite for the clinical application of such nanomaterials.^12^ Titanium dioxide nanoparticles, recognized for their chemical stability, environmental friendliness, and noncytotoxic nature, are regarded as intelligent drug delivery systems targeting specific microorganisms areas.

Their diminutive nanoscale dimensions make them particularly suitable for precision therapies, such as cancer treatment. In these systems, active substances adhered to nanomaterial-based carriers can selectively reach afflicted cells, bypassing healthy tissues and cells.^13,14^ This approach significantly enhances the therapeutic effectiveness while reducing the likelihood of adverse side effects. The assessment of titanium dioxide nanoparticles for their suitability in drug delivery systems hinges on various performance factors, including surface charge and the influence of pH on the release of active substances from the carrier’s body. Furthermore, their reception by cancer cells and cytotoxicity in target cells are subjected to scrutiny.^15^ Notably, titanium dioxide nanoparticles can transport different anticancer drugs on their surface. Moreover, these carriers themselves enhance the anticancer efficacy of these drugs, thanks to their unique properties.^16^ However, one should remember the possible dangers of using titanium dioxide nanoparticles as drug carriers. The toxic effects associated with titanium dioxide nanoparticles in humans primarily manifest as long-term consequences stemming from chronic exposure through various routes, including the respiratory, digestive, and dermal pathways.^17−19^ Titanium dioxide carriers may elicit toxicity toward organisms due to several factors. A primary concern arises from the capacity of titanium dioxide nanoparticles to provoke oxidative stress within cells, resulting in damage to cellular structures and biomolecules.^20^ Additionally, the small size and enormous surface area of titanium dioxide nanoparticles can facilitate cellular uptake, potentially perturbing cellular processes and inducing adverse effects.^21^ Furthermore, titanium dioxide nanoparticles may trigger inflammation and immune responses in organisms, exacerbating their toxicological impact.^22^ Moreover, the release of titanium ions from carriers can also contribute to toxicity, as elevated titanium ion levels may disrupt cellular functions and precipitate adverse health outcomes.^23^ The toxicity of titanium dioxide carriers toward organisms arises from their interactions with cellular constituents, leading to a spectrum of adverse effects at both cellular and organismal levels.

In Western countries, human exposure to titanium dioxide from everyday consumer products is estimated to be around 5 mg per person per day. For specific at-risk groups exposed to substantial quantities of these particles in the workplace (such as occupations involving paper bleaching, paint production, etc.) or those consuming products covered with these particles, these values may increase by roughly 10 to 100 times. Once titanium dioxide nanoparticles penetrate bodily tissues, they are not quickly removed and may accumulate over time. This accumulation can lead to extremely high levels after many years of exposure. Replicating such chronic exposures is exceptionally challenging, particularly in rodent models, as their lifespan is short and does not exceed two years. Consequently, most studies on the toxicity of these nanoparticles in animal models rely on different doses administered at one time or over a relatively limited time frame.^24^

The conclusions derived from scientific investigations concerning the influence of titanium dioxide on the functionality of specific organs within living organisms undeniably suggest that the utilization of nanoparticles as carriers for therapeutic agents is linked to adverse effects arising from their accumulation in human and animal tissues. Hence, the objective of this study was to modify titanium dioxide nanoparticles by depositing galactose molecules on their surface. It is assumed that the production of nanomaterials will be diversified by varying the parameters of the manufacturing processes. Additionally, the objective is to obtain nanocarrier–drug complexes (with tadalafil). The object was also to determine the physicochemical properties of the obtained materials and to demonstrate the limited release of metallic ions from the modified nanocarriers. Given that the developed technologies will exhibit characteristics of innovation, it is expected that the product recipients will demonstrate receptivity to modern solutions, thereby significantly enhancing their level of competitiveness. The ultimate beneficiary of the project outcome would be the patient, who will be protected from the harmful effects of drug carrier substances.

## 2 Materials and Methods

### 2.1 Materials

The modification process of titanium dioxide nanoparticles with galactose involved the use of the following compounds: titanium(IV) isopropoxide (TIPO) (97.0%), sodium hydroxide (≥98.5%), and d-(+)-galactose (≥99.0%). In the role of an active substance, tadalafil was used as a pharmaceutical secondary standard. For *in vivo* studies, the following chemicals were employed: ketamine, xylazine, buprenorphine, heparin, methanol, dimethyl sulfoxide, polyethylene glycol, physiological saline, formic acid, and acetonitrile. All compounds were sourced from Sigma-Aldrich. Also, male Wistar rats were used in the experiments. All aqueous solutions were prepared using deionized water (Polwater, 0.18 μS). For *in vitro* studies, culture media (F-12K medium), CHO cell line, and supplements (FBS, antibiotics) were utilized, all purchased from Sigma-Aldrich. The BrdU cell proliferation kit was purchased from Roche, and LDH cytotoxicity assay kit was purchased from Thermo Fisher Scientific.

### 2.2 Methods

#### 2.2.1 Process for Preparing of Modified Titanium Dioxide Loaded with Tadalafil

**Figure 1:**
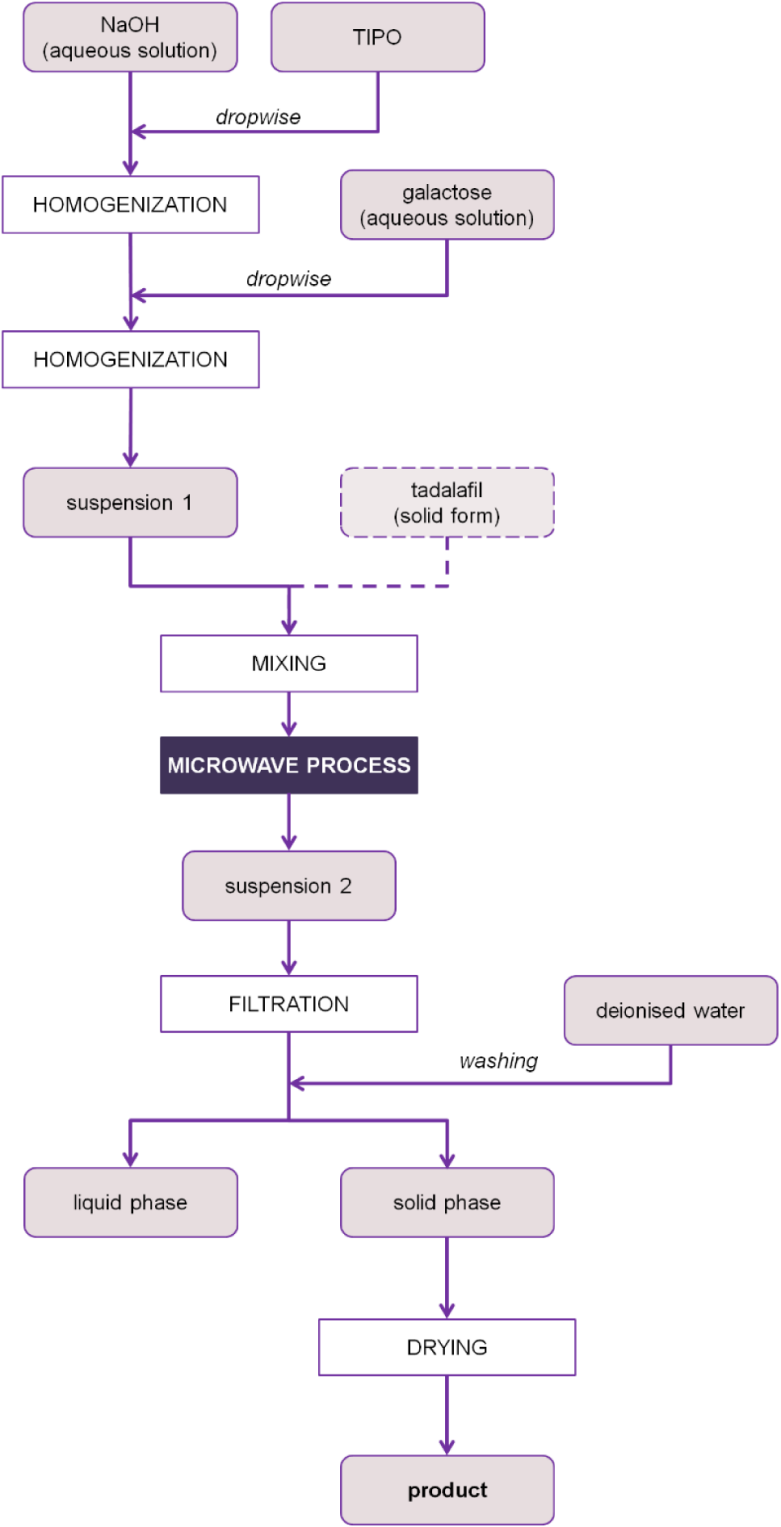
Schematic diagram ilustrating the process for preparing titanium dioxide with galactose as a modification agent and loaded with tadalafil.

Figure 1 shows the schematic diagram presenting the process of preparing titanium dioxide modified with galactose and with tadalafil as an active substance loaded. The amounts of all the reagents were calculated to obtain a final mass of titanium dioxide equivalent to 0.034 mol. In the first step, titanium(IV) isopropoxide was added in drops to an aqueous solution of sodium hydroxide, which was at a specific concertation. The amount of sodium hydroxide was adjusted so its fold vs stoichiometric amount required was equal to 1, 2, or 3 (as shown in Table 1). The mixture was homogenized in a Teflon vessel for 2 min (Hielscher UP400 St, Germany (40 W)) at ambient temperature. This step resulted in the formation of titanium hydroxide. Subsequently, an aqueous solution of galactose was added in drops and the created mixture was homogenized for another 2 min. The molar ratio of galactose to titanium dioxide varied, with values of 0.02, 0.11, or 0.2 (as shown in Table 1). Materials with incorporated tadalafil into their structure were also prepared. In this case, after the previous step a particular amount of powder tadalafil was put into the suspension 1, obtained in previous step. The resulting suspension was stirred on a magnetic stirrer (C-MAG HS 7, IKA) for an additional 5 min. The amount of tadalafil was calculated to achieve a mass ratio of 0.4 relative to titanium dioxide. The whole mixture was put into a microwave reactor (Magnum v2, Ertec, Poland), where the process of polycondensation of titanium hydroxide with water releasing and dehydration was performed. Nine products with varying process parameters were obtained. In addition, the process duration was another variable parameter, lasting for 2, 11, or 20 min (as outlined in Table 1). The process temperature remained constant at 150 °C. The resulting suspension 2 was filtrated using a Buchner funnel (with a pore size of 0.45 μm) and washed out with deionized water. After filtration, the filtrate was thrown away and the solid phase was put into a laboratory drier at 80 °C for 24 h. The reference sample, which was modified by depositing galactose, was prepared according to the same scheme.

**Table 1:** Processes Parameters

sample	*n*GAL/*n*TiO_2_	fold of NaOH vs stoichiometric amount	process time, min	process temperature, °C	*m*TAD, g
C1	0.02	1	2	150	1.079
C2	0.02	2	20		
C3	0.02	3	11		
C4	0.11	1	20		
C5	0.11	2	11		
C6	0.11	3	2		
C7	0.20	1	11		
C8	0.20	2	2		
C9	0.20	3	20		
C/Base	0	1	11		

The prepared materials underwent analysis to determine their physicochemical properties. X-ray diffraction (XRD) technique (Pert PW 1752/00 instrument from Philips) was used to reveal the crystallographic structure of titanium dioxide. Additionally, the presence of organic matter in the materials was confirmed using attenuated total reflection Fourier-transform infrared spectroscopy (ATR-FTIR) (Nicolet 380 spectrophotometer from Thermo Fisher). In order to asses the size of titanium dioxide, a dynamic light scattering (DLS) technique (Zetasizer Nano ZS from Malvern Instruments Ltd.) was applied. For this analysis, a series of suspensions containing the prepared products at a concentration of 10 mg/L was prepared and homogenized for 1 min prior to DLS analysis. Specific surface area, pore volume, and size were examined using low-temperature nitrogen sorption (ASAP2010 apparatus from Macromeritics USA). Before measurement, the samples were desorbed in a helium flow for 6 h at 200 °C and then under vacuum to reach a final pressure of 0.001 Torr. In order to asses the size and shape of the formed particles, transmission electron microscopy with an energy-dispersive X-ray spectroscopy (TEM-EDS) technique was employed (Tecnai TEM G2 F20X-Twin 200 kV from FEI).

#### 2.2.2 Assessment of Titanium Release from the Obtained Nanocarriers

To analyze the rate of titanium release from the prepared materials, elution studies in an aqueous environment were conducted. A specific amount of the material was weighted with analytical accuracy (0.15000 g) and introduced into a glass beaker. The appropriate volume of deionized water was then added to achieve a mass ratio of 0.05 between the nanocarrier and the eluting agent. To perform elution, the prepared suspensions were mixed using a magnetic stirrer at a constant temperature of 37 °C. The elution took 0, 1, 3, 5, 10, 20, 40, and 80 min. After each specific time point, the suspensions were passed through syringe filters (φ = 0.45 μm), and the titanium concentration in the filtrates was determined using atomic absorption spectrometry (PerkinElmer).

#### 2.2.3 Assessment of Tadalafil Release from the Obtained Materials

**Figure 2:**
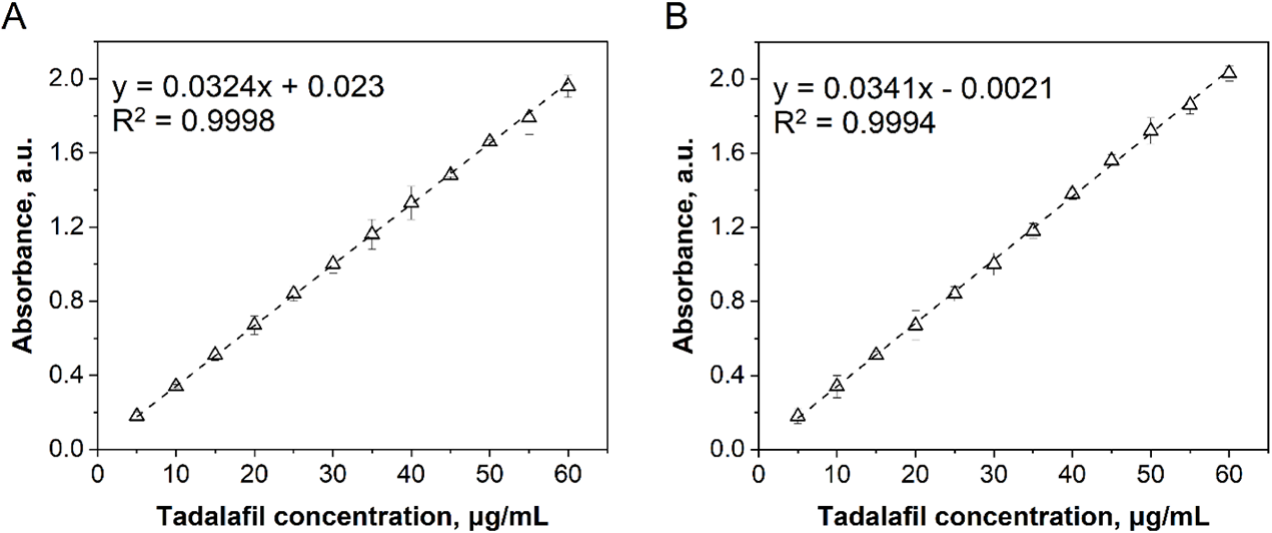
Calibration curves for the determination of tadalafil concentration in (A) Ringer solution and (B) SBF.

To assess the rate of release of active substance from the prepared materials, elution studies in various environments were conducted. For this, Ringer’s fluid or simultaneous body fluid (SBF) was added to a glass beaker containing a specific weight of the prepared material, with the mass ratio of 20:1 between the eluting agent and the material. The resulting suspensions were stirred using a magnetic stirrer at a constant temperature of 37 °C. After predetermined mixing intervals (0.5, 1, 3, 5, 10, 20, 30, 40, 50, 60, 120, and 180 min), the suspensions were passed through syringe filters (φ = 0.45 μm), and the tadalafil content in the filtrates was measured using UV–vis spectroscopy (Rayleigh UV-1800). The concentration of tadalafil was determined based on the calibration curves prepared at λ_max_= 284 nm (Figure 2).

#### 2.2.4 *In Vitro* Cell Viability Assay

In this study, we explored the effects of titanium dioxide nanoparticles, in both their unaltered and modified forms, on the cytotoxicity and proliferation of Chinese hamster ovary (CHO) cells. To analyze cytotoxicity, we used the lactate dehydrogenase (LDH) test.^25^ The cytotoxicity analysis was conducted in the following way: Chinese hamster ovary (CHO) cells were seeded in 96-well plates at a density of 9 × 10^3^ cells per well in 150 μL of medium. After a 48 h stabilization period, the culture medium was replaced with fresh medium that contained the tested nanocarriers. The nanoparticle concentrations in the suspensions were 10, 30, 50, 70, and 80 μg/mL, while the reference sample had no nanoparticles). Cytotoxicity was assessed using the Pierce LDH cytotoxicity kit (Thermo Fisher Scientific, Cat. No. 88954), and measurements were taken with a Multiskan GO microplate reader (Thermo Fisher Scientific) at two wavelengths: 490 nm (for formazan absorbance) and 680 nm (for background absorbance). The cytotoxicity was evaluated based on the equation:
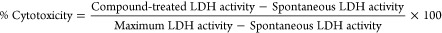


For the proliferation analysis, we used the BrdU assay.^26^ CHO cells were seeded in 96-well plates at a density of 9 × 10^3^ cells per well in 150 μL of medium. After a 24 h stabilization period, the culture medium was replaced with fresh medium containing the tested nanocarriers, with the control sample containing no nanoparticles. The cells were cultured an additional 48 h. The cell proliferation in the presence of the nanomaterials was measured using the cell proliferation ELISA kit for BrdU (Roche, Cat # 11647229001). Measurements were taken at two wavelengths: 450 nm (for materials absorbance) and 690 nm (for background absorbance) by using a Multiskan GO microplate reader (Thermo Fisher Scientific). To assess the DNA damage, the comet assay was performed.^27^ The extent of DNA damage was quantified by measuring the tail length and the amount of DNA it contained. CHO cells were seeded in 12-well plates at a density of 80,000 cells per well and cultured in 1 mL of medium for 24 h. The medium was then replaced with fresh medium containing the nanocarriers, and the cells were cultured for an another 24 h. After incubation, the cells were collected and suspended in 1% low melting point agarose (Eurx, Cat # E0303), which was then spread onto glass slides precoated with 1% agarose (Eurx, Cat # E0301). The slides were immersed in a lysis buffer (pH 10) containing of 2.5 M sodium chloride, 100 mM EDTA, 10 mM Trizma base, and 200 mM sodium hydroxide, for 1 h at 4 °C. They were then placed in an electrophoresis chamber with buffer (pH > 10) comprised of 300 mM sodium hydroxide and 1 mM EDTA, and electrophoresis was conducted at 18 V (0.5 V/cm) for 1 h. After electrophoresis, the slides were rinsed with distilled water and stained with SYBR Gold dye (ThermoFisher Scientific, Cat. No. S11494). DNA damage was detected using a ZOE Fluorescent Cell Imager fluorescence microscope, and the genotoxicity assessment was analyzed with CometScore 2.0 software.

#### 2.2.5 *In Vivo* Studies

**Figure 3:**
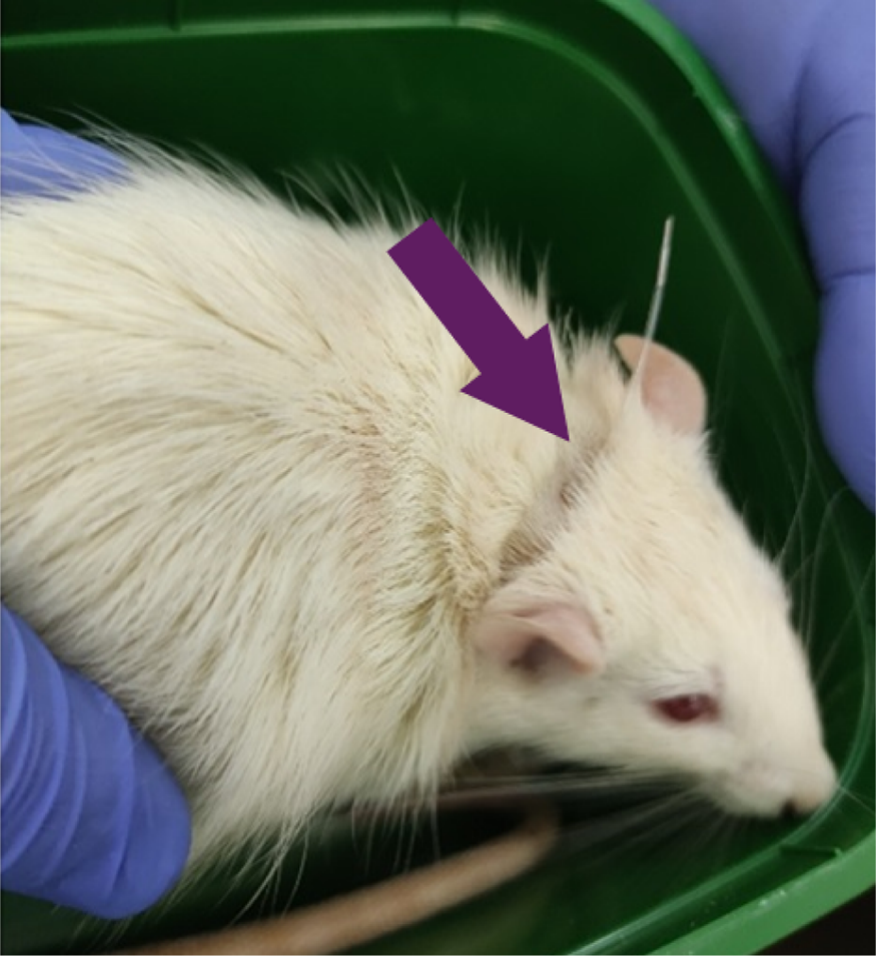
Rat cannulation site.

Male Wistar rats weighing approximately 300 g were used for the study. A rat jugular vein catheter (SAI Infusion Technologies, USA) was surgically inserted after incision of the body integuments, as shown in Figure 3. The experiment commenced 7 days postsurgery. The cannula was rinsed daily with heparinized saline.

**Figure 4:**
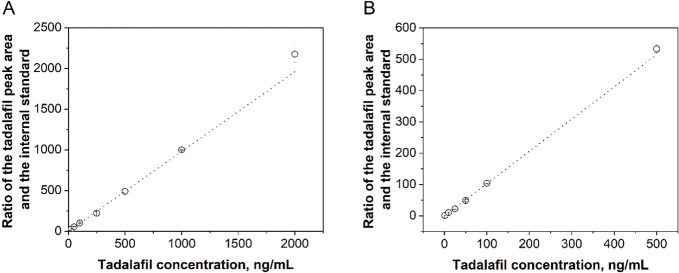
Calibration curves for the determination of tadalafil by the LC-MS method: (A) intravenous administration and (B) intragastric administration.

Tadalafil levels in the tested samples were measured by using high-pressure liquid chromatography with mass detection (HPLC/MS/MS). The analysis was conducted with a Sciex QTRAP 4500 triple quadrupole mass detector connected to an HPLC Excion LC AC system (Danaher Corporation, USA). The tadalafil analytical standard was prepared by dissolving the drug in methanol, whose concentration was equal to 1 mg/mL. Serial dilutions of this standard in methanol were used to create working standards with concentrations of 0.01, 0.1, 0.25, 0.5, 1, 2.5, 5, 10, and 20 μg/mL. Calibration samples were prepared by mixing 45 μL of pure plasma to 5 μL of the appropriate working standard concentration and vortexing for 10 s. The calibration curve was then plotted (Figure 4).

A series of solutions and suspensions were prepared for intravenous and intragastric administration of tadalafil. A 10 mg weight of tadalafil was dissolved in a mixture of 1 mL of dimethyl sulfoxide, 1 mL of polyethylene glycol (PEG 400), and 8 mL of water for injection and then filtered through a syringe filter. Three nanocarrier suspensions for intragastric administration were prepared by mixing 1 mL of dimethyl sulfoxide, 1 mL of polyethylene glycol, 6.8 mL of water for injection, and 1.2 mL of the appropriate nanocarrier containing tadalafil. The solutions were administered intravenously with a 0.5 mL needle into the caudal lateral vein, or intragastrically with a suitable probe, so that each animal received a dose of 1 mg/kg body weight.

After drug administration, blood samples were collected after 5, 15, 30 min, 1, 2, 4, 6, 8, 12, 16, and 24 h through a jugular catheter. A volume of 100 μL of blood was collected in heparinized tubes, followed by the administration of 100 μL of physiological saline after each sample was taken. Plasma samples (20 μL) were deproteinized by adding 80 μL (ratio 1:4) of 0.1% formic acid in acetonitrile with the addition of an internal standard and mixed for 10 min on a shaker (IKA Vibrax VXR, Germany). Subsequently, they were centrifuged at 8000*g* for 5 min (Eppendorf mini Spin centrifuge, Germany).

The serum concentration of tadalafil in the animal samples was determined by using the LC/MS/MS method. The Phoenix WinNonlin program (Certara, USA), was utilized to determine AUC_all_ parameters for intravenous and intragastric administration. The bioavailability of tadalafil was then calculated for the pure solution and tested nanocarrier suspensions relative to intravenous administration.

## 3 Results and Discussion

### 3.1 Modified Titanium Dioxide Loaded with Tadalafil

**Figure 5:**
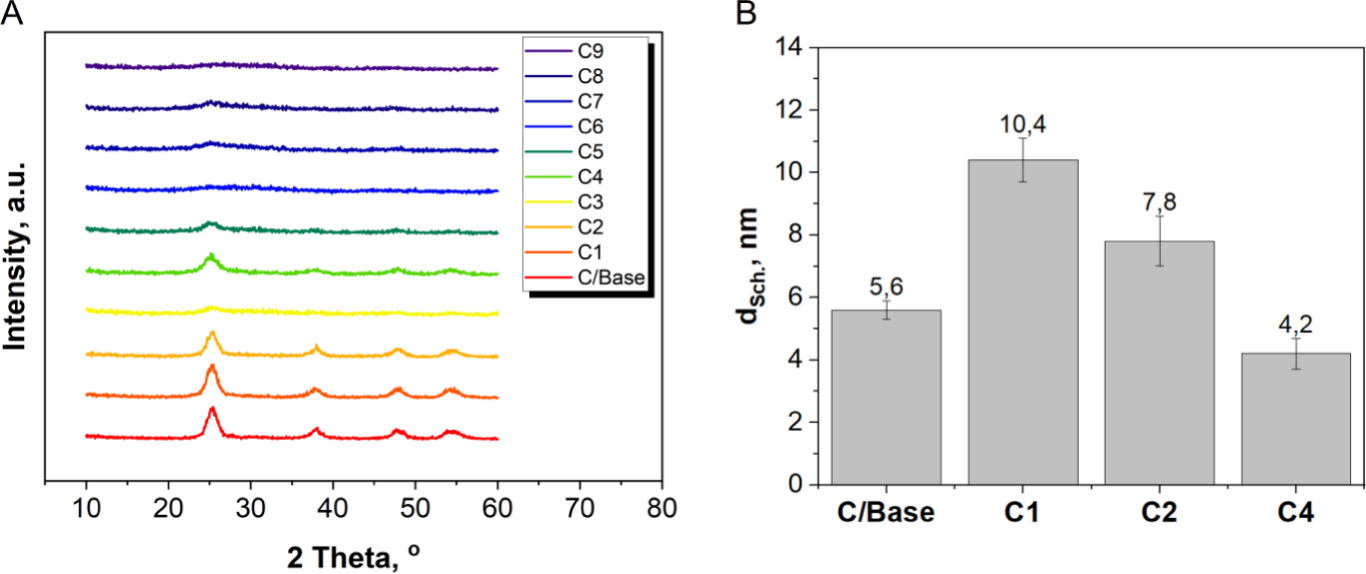
XRD patterns of all prepared materials (crystallite size calculated based on the Scherrer equation provided next to the material symbol).

According to the XRD findings (Figure 5A), it is apparent that not all of the products that were prepared exhibited a crystalline structure. Specifically, samples C/Base, C1, C2, and C4 displayed robust diffraction peaks at 25.30°, 37.90°, 47.80°, and 54.35° of the 2θ angle, which confirms obtaining of titanium dioxide in anatase phase (96-900-9087). The peaks were matched to the corresponding tetragonal crystal planes (101), (004), (200), and (105), respectively.^28−30^ The rest of the samples showed an amorphous structure. Crystalline structure was observed in the samples with the lowest or equal molar ratio of galactose to titanium dioxide (0.11) and an extended reaction time of 20 min (sample C4). Similarly, a crystalline structure was obtained when the fold of sodium hydroxide vs stoichiometric amount was the lowest or equal to 2, along with an extended reaction time of 20 min (sample C2). Aside from the peaks originating from titanium dioxide, no other peaks were observed, indicating the purity of the materials. The size of crystallites was determined using the Scherrer equation:^31^*d*_Sch_=*k*λ/βθcos θ

where *d*_Sch_ is the size of crystallites, *k* constant depends on the shape of the crystallite size, β is the width at half-maximum peak describing the material, λ is the wavelength of Cu Kα radiation, θ is the Bragg diffraction angle. Figure 5B displays the analysis results, showing that all crystallites are either below or approximately. This indicates a substantial increase in specific surface area. However, it is noteworthy that the unmodified base sample (C/Base) has smaller crystallite sizes compared to materials C1 and C2. This suggests that the modifier molecules may block the pores, thus limiting the expansion of the surface area.

**Figure 6:**
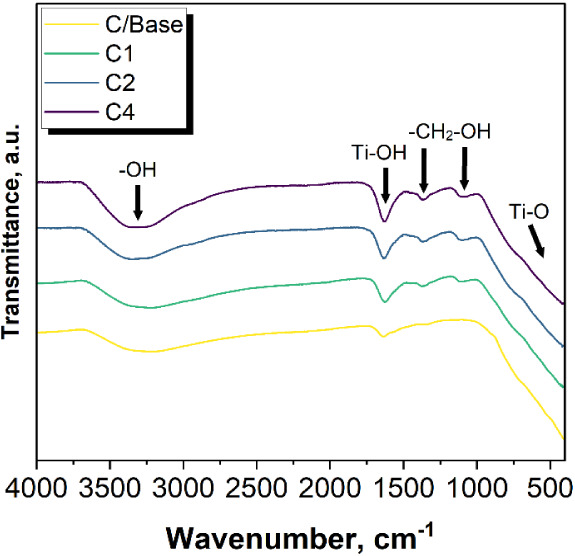
FTIR spectra of obtained materials.

Results of the FTIR analysis are presented in Figure 6. Based on the spectrum, one may conclude that titanium dioxide has been confirmed in all the analyzed samples. Ti–O bending is observed at 500 cm^–1^. The deformative vibration of the Ti–OH stretching mode may be seen at 1623 cm^–1^, attributable to the water adsorbed on the surface of titanium dioxide. The pronounced peak at approximately 3320 cm^–1^ is associated with the asymmetrical and symmetrical stretching vibrations of hydroxyl group.^32^ The presence of galactose is confirmed by peaks at 1363 and 1110 cm^–1^, which correspond to −CH_2_–O stretching vibrations.^33^

**Figure 7:**
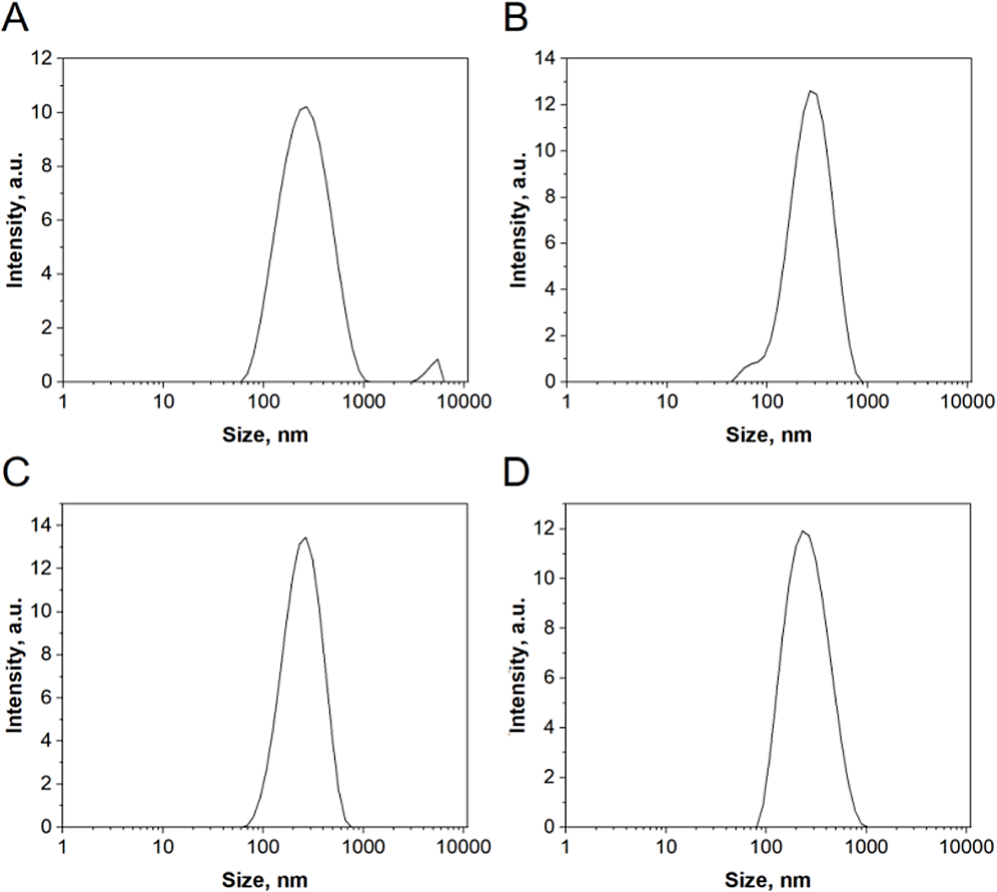
Results from the DLS analysis displaying nanoparticle sizes (A) C/Base, (B) C1, (C) C2, and (D) C4.

The DLS technique was used to measure the hydrodynamic size of nanoparticles, denoting the apparent dimensions of particles or molecules in a liquid environment, as they undergo random movement due to Brownian motion. This measurement considers both the shape and size of the nanoparticles as well as the influence of surrounding solvent molecules. It provides insights into the observable size of particles in a solution, taking into consideration their diffusion characteristics.^34^ The results are depicted in Figure 7. The dominant particle size of the reference particles was 311 nm, but larger particles measuring up to 4700 nm were also present. The presence of such large particles may be attributed to the absence of a stabilizing factor, allowing them to grow. In the case of galactose-modified particles, the sizes in materials C1 and C2 were similar, at 310 nm. In sample C2, smaller particles of approximately 90 nm were also present. In material C4, the influence of an increased amount of the modifying agent on the titanium dioxide particle size is noticeable, as it measured at 268 nm. Most anticancer drugs are small, typically less than 10 nm in size. Using them unchanged would result in their diffusion into healthy and diseased tissues equally. However, when combined with nanocarriers (ranging from 50 to 800 nm), the penetration of these drugs into healthy tissues is significantly reduced or even eliminated.^35^ Taking this into account, the obtained results are very satisfactory, as the size of the nanoparticles is in the desired range.

**Figure 8:**
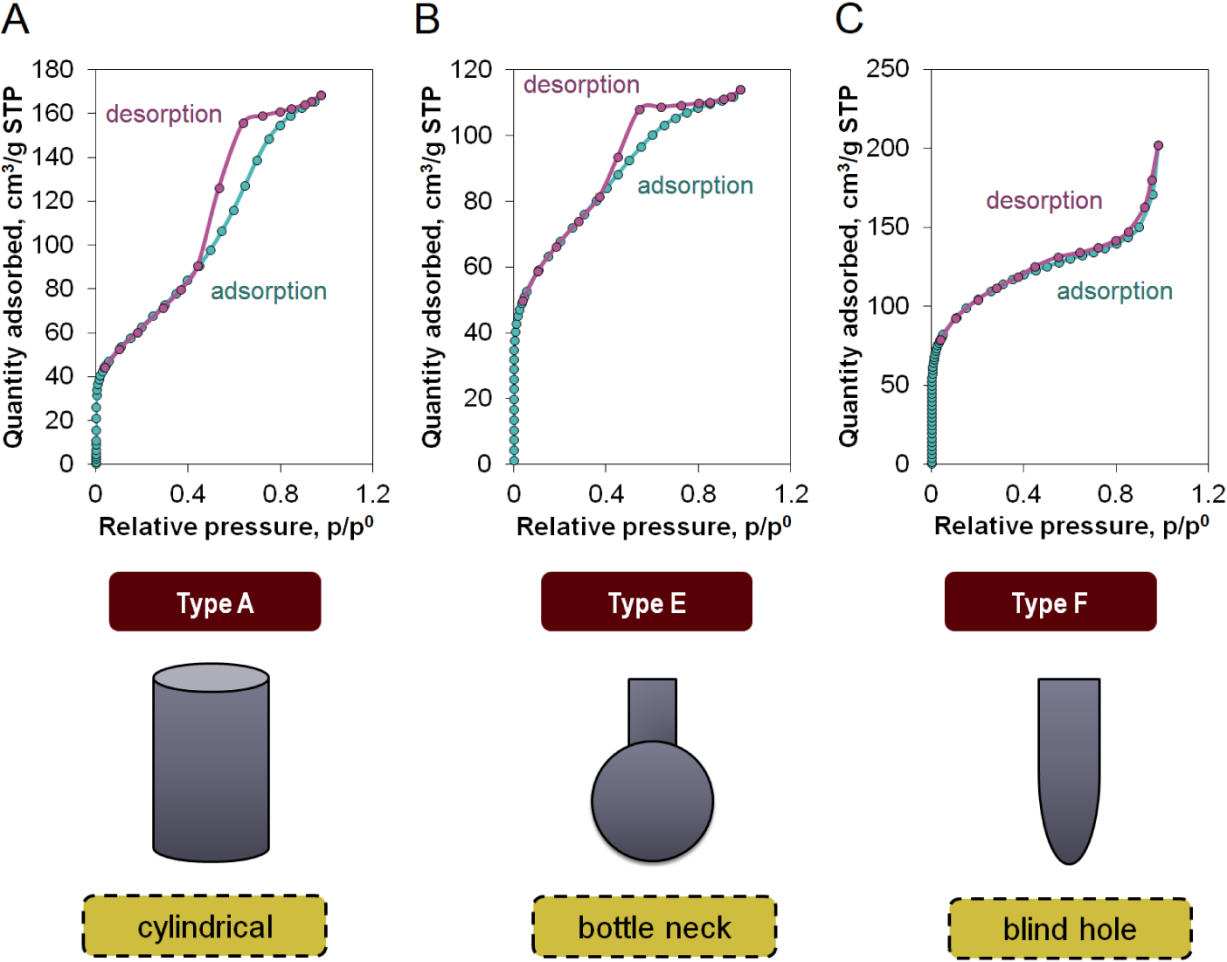
Hysteresis loops with identified shapes of pores: (A) C/Base, (B) C2, and (C) C4.

Figure 8 shows the hysteresis loops and identifies pore types based on these characteristics. The hysteresis loop types can be correlated to the shape of the pores. It can be observed that each of the materials examined exhibited a distinct type of hysteresis loop and, consequently, different pore shapes. This diversity was influenced by variations in the process parameters. The reference material (C/Base) featured cylindrical pores, indicated by the type A hysteresis loop. Sample C2 was characterized by a type E hysteresis loop and bottleneck-shaped pores. Meanwhile, material C4 had blind hole pores, as evidenced by the type F of hysteresis loop.^36^ The measured specific surface area and surface parameters provided in Table 2 indicate a high surface area development in the obtained materials, making them suitable for use as carriers for active substances.

**Table 2:** Results of Analysis of Surface Parameters

sample	SS, m^2^/g	V, cm^3^/g	size, nm
CB	228.9	0.4842	1.0650
C2	234.0	0.4157	0.7847
C4	355.4	0.6335	0.8790

**Figure 9:**
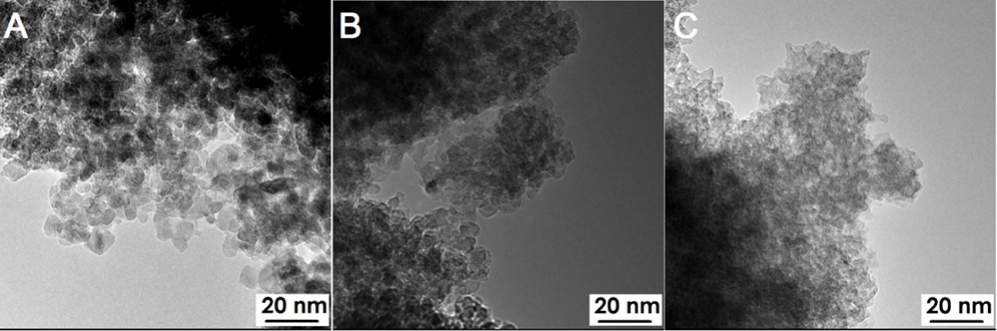
TEM microphptographs: (A) C/Base, (B) C2, and (C) C4.

The results of TEM analysis are presented in Figure 9. Material C4 has the smallest particles, around 5 nm, which is consistent with the surface property analysis results. Both the reference product and material C2 contain titanium dioxide nanoparticles no larger than 10 nm. In the images, the particles are fairly well separated from each other with a visible envelope. Unfortunately, the particle size determined from the TEM photographs does not correlate with the particle size measured by the DLS technique. However, this does correspond to calculations regarding crystallite sizes. It is worth noting that the DLS technique is based on Mie theory, which assumes a spherical particle size.^37^ Due to the lack of a stabilizing substance, the particles could agglomerate during the measurement, potentially leading to distorted and falsely increased DLS measurement results.

**Figure 10:**
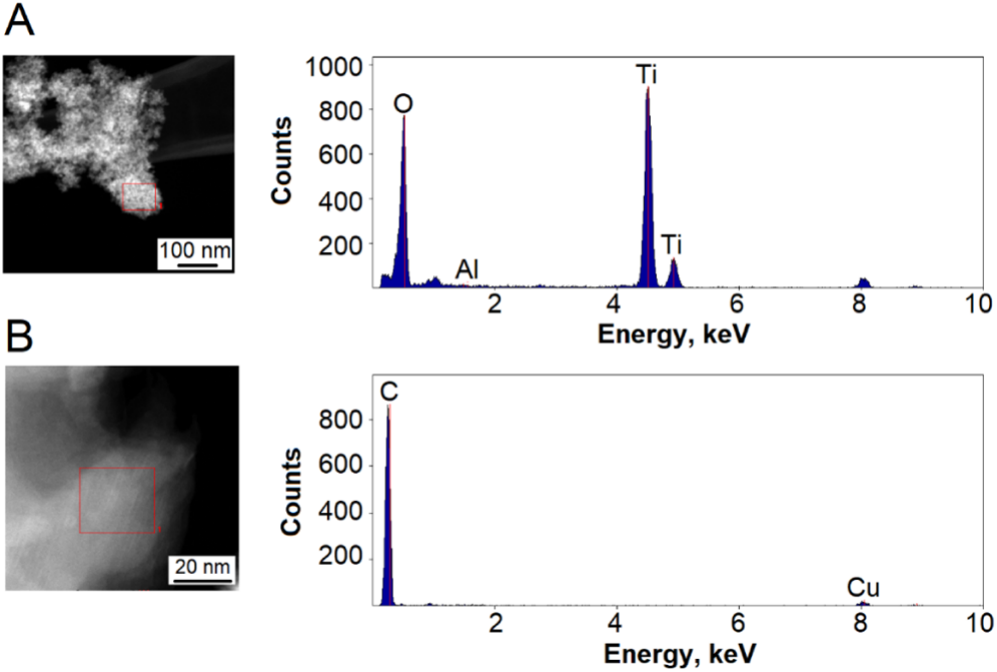
Results of the TEM-EDS analysis: (A) C/Baseand (B) C2.

The TEM-EDS analysis results are displayed in Figure 10. Pure, unmodified titanium dioxide nanoparticles consist solely of titanium and oxygen, as shown in Figure 10A. This analysis reveals that the halo surrounding the titanium dioxide nanoparticles is composed of carbon, originating from organic matter provided by galactose (Figure 10B). Any inclusions of other elements (aluminum and copper) came from the background of the equipment.

### 3.2 Titanium Elution from the Prepared Complexes

**Figure 11:**
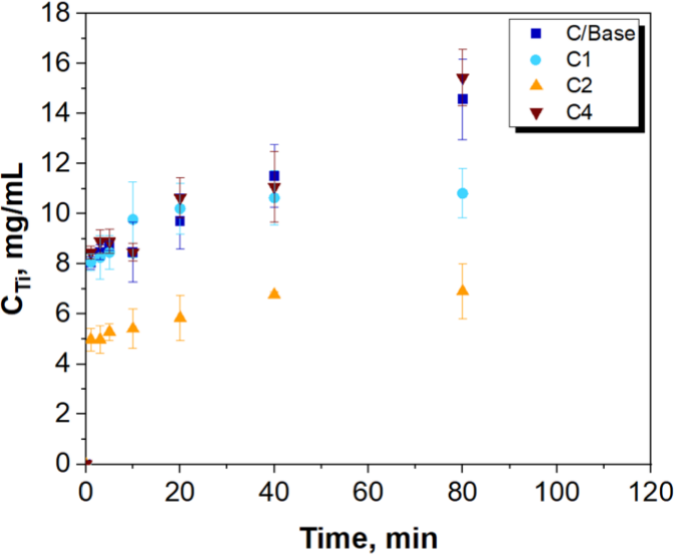
Profiles of titanium released from the prepared materials.

The analysis results for titanium release from the prepared materials are presented in Figure 11. One of the main goals of this study was to minimize titanium leaching, as the release of titanium, in either metallic or ionic form, poses a potential risk to living organisms. These forms of titanium can accumulate in tissues and potentially promote tumor development. Therefore, preventing the release of metals from drug carrier systems is crucial. In the figure, the navy blue squares represent the titanium release profile from the reference sample (unmodified). As can be seen, the release of titanium from the C2 material is the lowest and stabilizes over time at a constant level (approximately 7 mg/mL after 40 min). The C1 material also has a satisfactory release profile. After 40 min, the titanium concentration in the eluting medium also stabilizes and is lower than in the case of the reference sample (bare titanium dioxide). The concentrations of titanium in the eluting medium tested in the case of the analysis of C4 and reference samples are similar and continuously increase over time. Material C2 was obtained when *n* GAL:*n* TiO_2_ was equal to 0.02, the fold of sodium hydroxide vs stoichiometric amount was equal to 2, and process time was equal to 20 min. These process parameters mean that galactose encapsulating titanium dioxide effectively limits the release of titanium ions from the material.

The primary purpose of using coating compounds is to enhance nanoparticle stability by preventing ion leaching, protecting against surface oxidation, and reducing nanoparticle aggregation and agglomeration. Huang et al. demonstrated that applying a thin silica dioxide layer as a coating for AgNPs effectively reduced their toxicity by obstructing ion release and contact with bacteria and/or cells.^38^ Therefore, it seems to be a good idea to encapsulate metal oxide nanoparticles to prevent the release of toxic ions from them.

### 3.3 Tadalafil Elution from the Prepared Complexes

**Figure 12:**
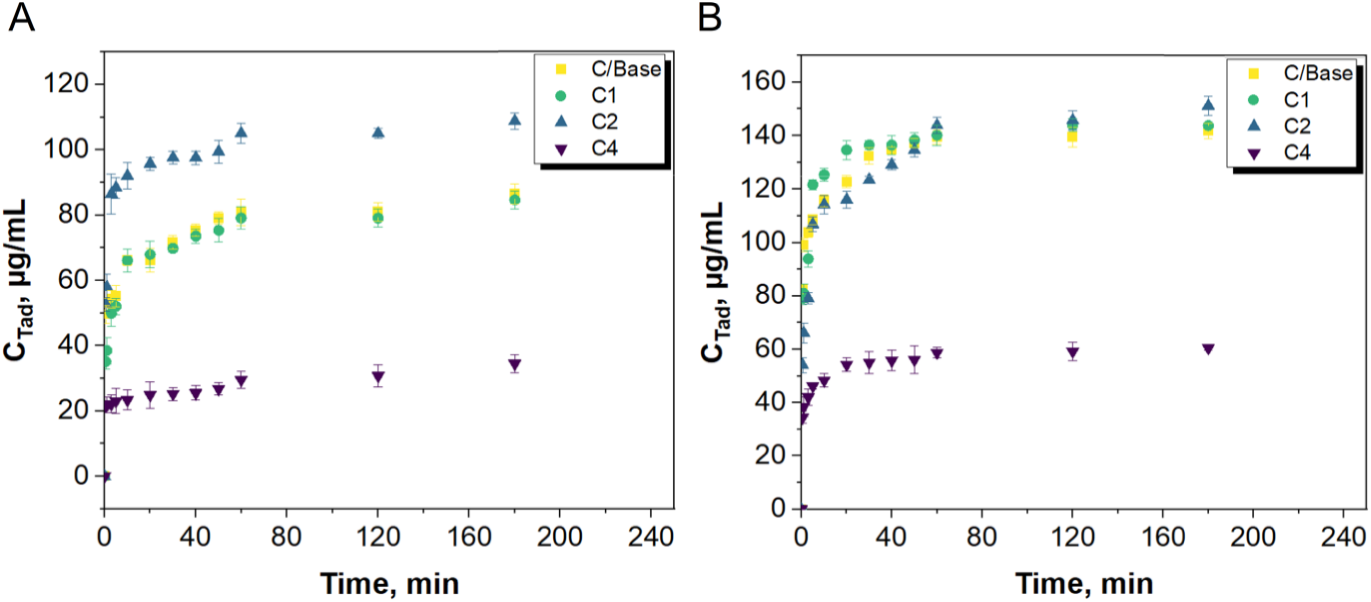
Profiles of the tadalafil elution: (A) Obtained in Ringer’s fluid and (B) obtained in SBF.

Figure 12 presents the results of tadalafil elution analysis. Yellow squares present the elution profiles from reference material (not modified titanium dioxide). Analysis performed with using Ringer’s fluid as an eluting medium confirmed that the lowest share of the released active substance was observed in the case of material C4, then for both material C1 and the reference sample, and the highest concentration over time was observed for sample C2. When the elution medium was SBF, it was observed that the lowest share of the released active substance also occurred in the case of material C4, and the concentrations of tadalafil for the C/Base, C2, and C1 samples were similar over time. Various established mechanisms govern the release of the active substance from the transport system.^16^ The release behavior depends on both the stability of the active substance and the physicochemical properties of the nanocarrier. For traditional polymeric drug carriers, drug release profiles typically follow the degradation kinetics of the biodegradable polymers. The release kinetics of polymer nanoparticles, such as poly(lactic-*co*-glycolic acid), can be finely tuned by adjusting the composition of the polymeric shells, including the lactide/glycolide ratio and molecular weight.^39^ However, for stable solid drug carriers, such as metal oxide nanoparticles, the release kinetics are more complex. The key components of the release process include: (1) detachment of surface-bound drug molecules, (2) diffusion of the drug away from the carrier’s surface, (3) erosion of the carrier itself, and (4) an interplay of erosion and diffusion processes.^40^ The release profiles adhere to the characteristic diffusion profile commonly observed in the nanoparticle-based drug carriers, as indicated in reference.^41^ The diffusion mechanism is suitable for systems where the drug’s diffusion rate surpasses the carrier’s degradation rate, a characteristic that aligns with the inherent nature of the carrier employed in this study, specifically a metal oxide-based carrier. The studies revealed an initial rapid release, commonly referred to as a “bursting release”, followed by a subsequent “sequential” release.

This release profile is attributed to complexes in which the drug is adsorbed or weakly bound to the carrier surface. When simulated body fluid (SBF) was used as the receiving medium, the eluted tadalafil concentration was higher than when Ringer’s solution was used, consistent with expectations. The rate of drug release can be influenced by ionic interactions between the carrier and other components of the receiving medium. The composition of SBF is more complex than the Ringer’s solution. In the SBF environment, competitive electrostatic interactions between the carrier and surrounding ions reduce the interaction of the active substance with the carrier matrix, explaining the increased drug release in this setting.

## 4 *In Vitro* Cell Viability Assay

**Figure 13:**
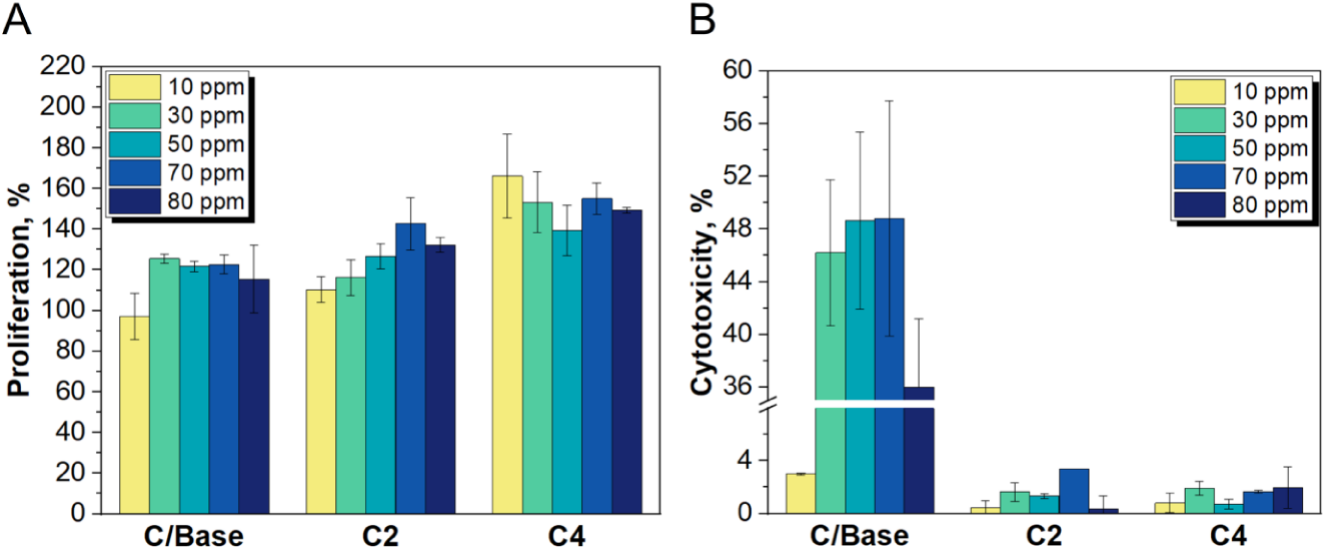
(A) Proliferation analysis results and (B) Cytotoxicity analysis results.

The effects of the *in vitro* cell viability analysis are shown in Figure 13. In Figure 13A, the relationship between the cytotoxicity and the concentration of the analyzed materials is illustrated. It is evident that all the tested materials modified with galactose elicited a more robust proliferation of CHO cells compared to the reference sample, which was not modified and consisted of basic titnaium dioxide. Interestingly, titanium dioxide nanoparticles modified with galactose even stimulated the growth of CHO cells. Additionally, when the cytotoxic properties of modified materials C2 and C4 are compared to the reference material (Figure 13B), it is evident that the modified materials displayed significantly lower cytotoxicity. This result is due to the presence of galactose on their surfaces, which effectively prevented the release of Ti ions that could lead to the formation of reactive oxygen species (ROS) and subsequent cell apoptosis.

**Figure 14:**
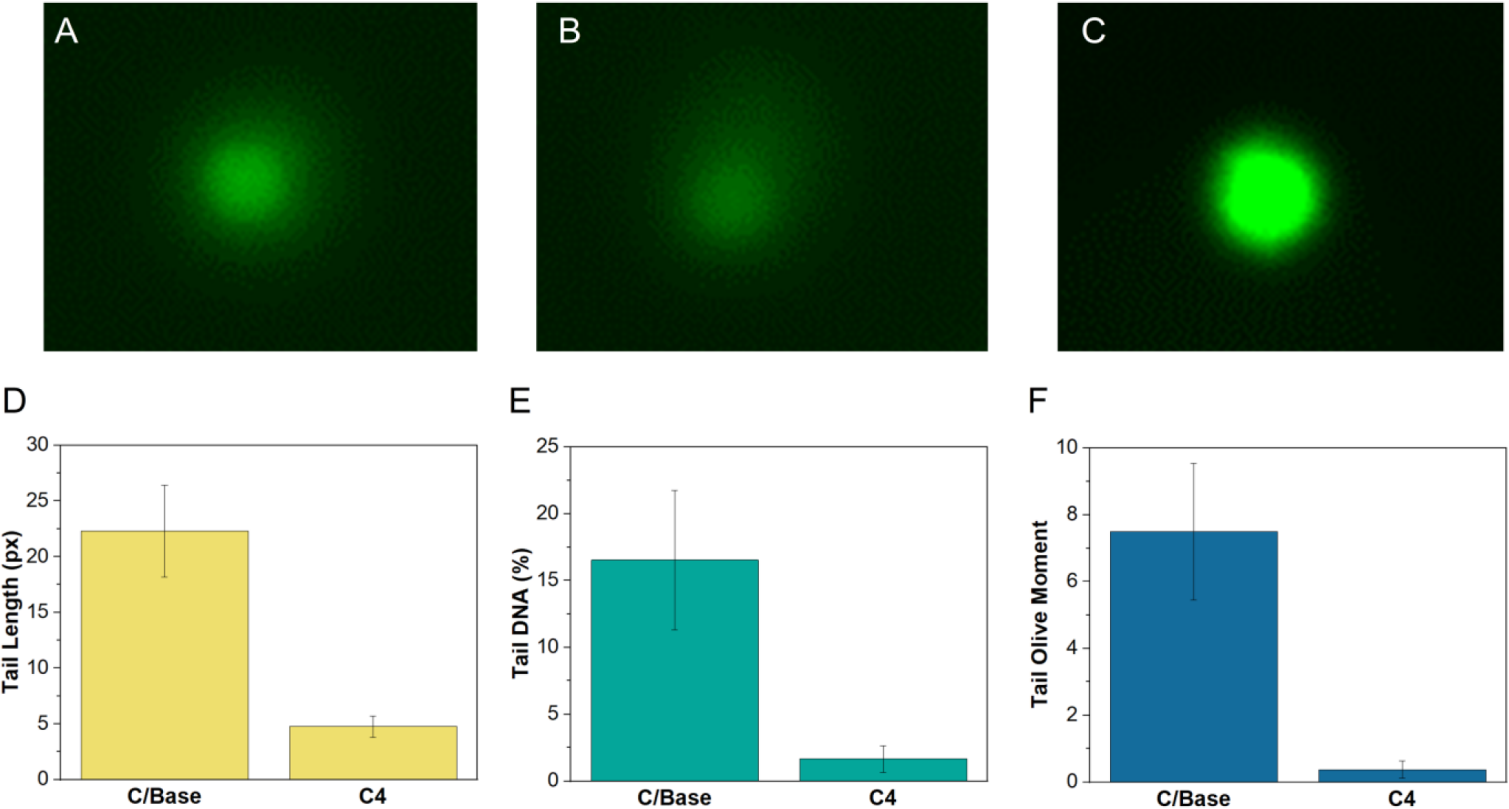
Comet test results: (A) Positive control, (B) C/Base, (C) C4, (D) tail length, (E) DNA tail and (F) olive moment.

Compared to the reference material, which consists of unmodified nanoparticles, the tested products produced shorter comet tails, indicating a reduced level of cell apoptosis, as shown in Figure 14. This finding suggests that the modified titanium dioxide nanoparticles cause less DNA damage, resulting in lower genotoxicity and mutagenicity. These observations are quantified in Figure 14 D–F, where all the tested parameters (tail length, DNA tail, olive moment) for the reference material consistently exceed those for the modified titanium dioxide. This aligns with the study’s objectives, demonstrating a reduction of genotoxic and mutagenic properties in the tested materials.

Various methods can mitigate or reduce the toxic effects of metallic nanoparticles and metal oxides. Research suggests that modifying the physicochemical properties, such as shape and size of these particles, along with employing surface modification techniques, can produce nanoparticles with desired properties while avoiding toxicity.^42^ Korábková et al. conducted experiments exposing titanium dioxide particles in both rutile and anatase forms, as well as their trade mixtures, to various environments. These included simulated gastric fluids and human blood plasma, which corresponded to *in vivo* conditions, and media commonly used in *in vitro* experiments. The authors utilized SBF with varied compositions, ionic strengths, and pH levels to study the effects of specific enzymes’ presence or absence. The aim was to determine the physicochemical properties and agglomeration behavior of titanium dioxide within these diverse media. They observed that the type of titanium dioxide and the surrounding environment significantly influenced the time-dependent agglomeration of titanium dioxide. Moreover, the presence of enzymes had a contrast varying effect, either inhibiting or promoting titanium dioxide agglomeration. Besides agglomeration dynamics, titanium dioxide showed a concentration-dependent cytotoxicity. Understanding titanium dioxide behavior in all these environments is crucial for assessing its safety, especially considering the significant impact of protein presence and size-related cytotoxicity.^43^ Hamzeh and Sunahara studied the cytotoxicity and genotoxicity of commercially available titanium dioxide nanoparticles, focusing on their specific physicochemical properties and the effects of surface coatings. They found that all the tested titanium dioxide samples reduced cell viability in concentration- and size-dependent manner. Notably, polyacrylate-coated titanium dioxide nanoparticles exhibited cytotoxicity at higher concentrations. A similar trend was observed for the induction of apoptosis/necrosis with no DNA damage detected in the polyacrylate-coated nanoparticles. Considering the growing production of titanium dioxide nanoparticles, it is essential for toxicological studies to consider these nanoparticles’ physicochemical properties. This approach can help researchers develop new nanoparticles with minimized toxicity.^44^ Hanot-Roy et al. investigated the impact of titanium dioxide nanoparticles on cell lines representative of the alveolo–capillary barrier. They found that all cell lines exposed to nanoparticles generated reactive oxygen species (ROS). Macrophage-like THP-1 and HPMEC-ST1.6R microvascular cells were sensitive to endogenous redox fluctuations, leading to apoptosis, whereas alveolar epithelial A549 cells did not exhibit apoptosis. The genotoxic potential of titanium dioxide nanoparticles was evaluated by assessing γH2AX, DNA repair protein activation, and cell cycle arrest. In sensitive cell lines, persistent DNA damage and the activation of DNA repair pathways were observed. Additionally, Western blot analysis revealed the simultaneous activation of specific pathways related to cellular stress responses, in tandem with DNA repair or apoptosis. Oxidative stress induced by nanoparticles acts as a critical signal transducer for subsequent physiological effects, including genotoxicity and cytotoxicity. Within these activated pathways, HSP27 and SAPK/JNK proteins emerged as potential biomarkers of intracellular stress and sensitivity to endogenous redox fluctuations.^45^

## 5 Results of *In Vivo* Analysis

**Figure 15:**
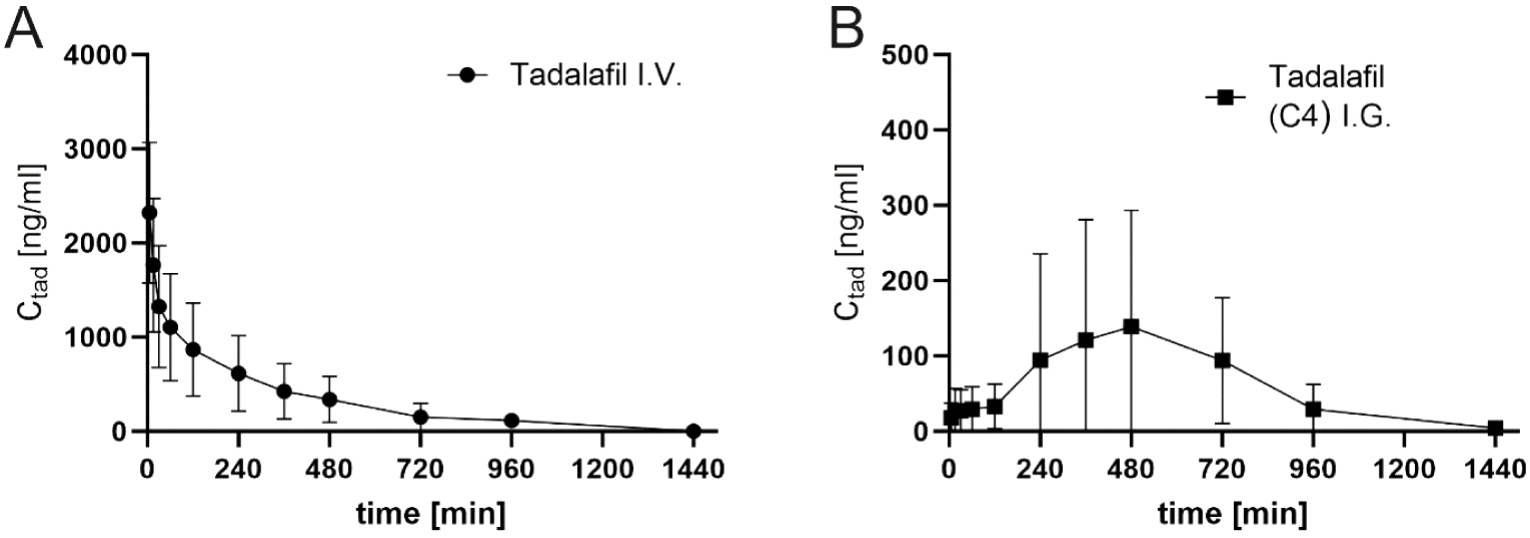
Tadalafil release profiles after (A) intravenous or (B) intragastric administration with the C4 nanocarrier. Data expressed as mean (±SD), *n* = 4–5.

Figure 15 shows the results of serum sample analyses after intravenous and intragastric administration of tadalafil. Table 3 shows the calculated pharmacokinetic parameters and bioavailability.

**Table 3:** Values of Areas under the Curve and Bioavailability of Tadalafil After Intravenous and Intragastric Administration Without and With the C4 Nanocarrier^a^

nano carrier symbol	mean AUC_all_ [min × ng/mL]	mean AUC_inf_ [min × ng/mL]	mean bioavailability [%]
I.V. Pure Tadalafil	456222.63	456877.56	-
I.G. Tadalafil with C4 nanocarrier	90350.204	91363.129	19.80397246

a Bioavailability calculated as AUC_all_ I.G./ AUC_all_ I.V.

Based on the values of the areas under the concentration–time curve calculated up to the last measured time point (AUC_all_) and infinity (AUC_inf_), bioavailability of tadalafil after intragastric administration in suspensions containing C4 nanocarrier was calculated. The modified nanocarrier showed improved bioavailability (19.8%), but also increased a spread of concentrations in the tested time points. The pharmacokinetic parameters for this material in the analysis of bioavailability of an active substance were as follows: *C*_max_ was equal to 138.9 ng/mL and *t* for *C*_max_ was equal to 480 min.

This study primarily aligns with the criterion of product innovation, although elements of process innovation are also evident. The process operates within the domain of microwave radiation, demonstrating superior efficiency compared to conventional methods of heating reaction mixtures. Microwave radiation generates thermal energy in a more effective way, presenting a significant advantage. In this innovative approach, aqueous solutions containing all the reactants are utilized. Water, as a polar solvent, is an optimal medium that quickly and efficiently facilitates the transfer of thermal energy induced by the microwave field. Through dipole polarization, heat is evenly distributed throughout the reaction mixture. Notably, the application of microwave energy substantially accelerates chemical reactions, leading to faster reaction kinetics and reduced reaction times. Under microwave radiation, a temperature of 150 °C is reached, which is suitable for the polycondensation of titanium hydroxide and the dehydration of the condensation product to titanium dioxide and water. An inherent benefit of this method is the environmentally friendly nature of the reagents used. They do not irritate or harm living organisms, particularly the modifying agent galactose. Additionally, the process does not produce solid waste, eliminating the need for waste management measures.

## 6 Conclusion

A series of titanium dioxide nanoparticles modified with galactose were meticulously prepared. Among the various materials studied, material C4 emerged as the prime candidate meeting all of the predetermined criteria. The criteria included both physicochemical and application properties. In particular, it was necessary to obtain a formulation that is stable, and its particle size does not exceed 800 nm. In addition, from the point of view of the possibility of loading the drug carrier with the active substance, its highly developed specific surface area is important, which was met. Modification of the drug carrier with galactose resulted in a favorable effect on the proliferation of CHO cells and caused a reduction in cytotoxicity and mutagenicity compared with the reference material. It was also important that the modified carrier exhibited a favorable release profile of the active substance *in vivo* studies.

The most desired nanocarrier was synthesized under the following conditions: *n* GAL:*n* TiO_2_ was equal to 0.11, the fold of NaOH was stoichiometric and it was equal to 1, process time was 20 min, and process temperature was 150 °C. The preparation of this material holds significant promise for further investigation as a robust solid drug carrier.

## Supporting Information Available

Additional methods details, including description of *in vitro* cell viability assay and *in vivo* studies (PDF)
