# Supplementary material for: In Vitro and In Vivo Studies of Titanium Dioxide Nanoparticles with Galactose Coating as a Prospective Drug Carrier
Source: ACS Omega. 2024 Aug 15;9(34):36220–31. doi: 10.1021/acsomega.4c02232 (PMC11360011; doi:10.1021/acsomega.4c02232)
Supplement: Supplementary file 1 — ao4c02232_si_001.pdf [file ao4c02232_si_001.pdf]

In vitro and *In vivo* studies of titanium dioxide nanoparticles with galactose coating as a prospective drug carrier

Jolanta Pulit-Prociak[1]*, Olga Długosz[1], Anita Staroń[1], Dominik Domagała[2], Krzysztof Pociecha[3], Mikołaj Grabowski[1], Michał Zielina[4], Marcin Banach[1]

[1]Faculty of Chemical Engineering and Technology, Cracow University of Technology, Warszawska 24, Cracow 31-155, Poland
[2]Faculty of Food Technology, University of Agriculture in Krakow, Balicka 122, Cracow 30-149, Poland
[3]Faculty of Pharmacy, Jagiellonian University, Medyczna 9, Cracow 30-688, Poland
[4]Faculty of Environmental Engineering and Energy, Cracow University of Technology, Warszawska 24, Cracow 31-155, Poland

E-mail addresses:
Jolanta Pulit-Prociak – jolanta.pulit-prociak@pk.edu.pl
Olga Długosz – olga.dlugosz@pk.edu.pl
Anita Staroń – anita.staron@pk.edu.pl
Dominik Domagała – domagala.ur@gmail.com
Krzysztof Pociecha – k.pociecha@uj.edu.pl
Mikołaj Grabowski - mikteograb@gmail.com
Michał Zielina – michal.zielina@pk.edu.pl
Marcin Banach – marcin.banach@pk.edu.pl

*Corresponding author:
Jolanta Pulit-Prociak – jolanta.pulit-prociak@pk.edu.pl
Faculty of Chemical Engineering and Technology,
Institute of Chemistry and Inorganic Technology,
Cracow University of Technology,
Warszawska 24
Cracow 31-155
Poland
phone: +48126282092
fax: +48126282035

Commented [J1]: E/1:
The first page of the Supporting Information has been added and it matches the manuscript title page.

1.1.4. *In Vitro* Cell viability assay

The following materials were employed in the investigation: CHO cells (Sigma-Aldrich, catalog no. 85051005), cultured according to the manufacturer's guidelines; F-12K medium (Sigma-Aldrich, catalog no. N4888) supplemented with fetal bovine serum (Thermo Fisher Scientific, catalog no. 10270106) and antibiotics (Sigma-Aldrich, catalog no. P4333). The cultures under scrutiny were maintained at 37°C with a 5% CO_2 atmosphere, and cell passaging occurred when they reached 80% confluence, typically 2-3 times per week. The LDH test enabled the quantification of formazan formation from tetrazoline, measured by absorbance at 490 nm. This reaction is facilitated by lactate dehydrogenase in the presence of NAD+. When cell membranes are broken, lactate dehydrogenase is released from dead cells into the culture medium.[25]

The BrdU proliferation analysis assay is a colorimetric method used to quantify the incorporation of 5-bromo-2'-deoxyuridine (BrdU) into DNA. BrdU is a synthetic analog of thymidine nucleoside and is integrated into the DNA of dividing cells during the S phase of the cell cycle. To assess the level of BrdU incorporation, an enzymatic reaction involving an enzyme-conjugated with an anti-BrdU antibody and the substrate is conducted in previously fixed cells. A low signal suggests a suppression of cell division, whereas a high signal indicates robust proliferative activity in the cells.[26]

The comet assay is a technique used to identify DNA damage at the individual cell level. In this method, the cells under examination are embedded in agarose on a microscope slide. After the proteins are digested, the DNA remains intact. The slide is then subjected to electrophoresis and subsequently stained with a fluorescent substance, resulting in the formation of distinctive "comets" on the slide. These comets consist of a "head," representing the region where the cell was immobilized before lysis, and a "tail," comprising the damaged DNA fragments.[27] The extent of DNA damage is quantified by measuring the length of the tail and the amount of DNA it contains.

1.1.8. *In Vivo* studies

The animals were kept in the Animal House of the Faculty of Pharmacy of the Jagiellonian University Medical College in Cracow. Rats were housed indoors at 21°C, in 50% atmospheric humidity, and a 12-hour lighting cycle. The animals acclimated to the conditions in the room where they remained during the experiment for 5 days. Before starting the research, the consent of the Local Ethical Committee was obtained for the experiment on animals (resolution number: 171/2018). The animals were subjected to general anesthesia by intraperitoneal administration of a solution of ketamine with xylazine (50/7.5 mg/kg b.w.). After depilation of the right shoulder and neck area, a rat jugular vein catheter (SAI Infusion Technologies, USA) was inserted after incision of the body integuments. The cannula was inserted into the rat's right common jugular vein, tied with surgical threads, and led out around the nape of the neck. The incision site was sutured with surgical staples and the animal was allowed to awake. On the first day after surgery, buprenorphine was administered as a subcutaneous injection every 8 hours.
